# Porous Polymeric Microspheres With Controllable Pore Diameters for Tissue Engineered Lung Tumor Model Development

**DOI:** 10.3389/fbioe.2020.00799

**Published:** 2020-07-10

**Authors:** Dinesh Dhamecha, Duong Le, Rachel Movsas, Andrea Gonsalves, Jyothi U. Menon

**Affiliations:** Department of Biomedical and Pharmaceutical Sciences, College of Pharmacy, University of Rhode Island, Kingston, RI, United States

**Keywords:** controlled pore formation, scaffold, tissue engineered, drug screening, microparticles, lung cancer co-culture

## Abstract

Complex cell cultures are more representative of *in vivo* conditions than conventionally used monolayer cultures, and are hence being investigated for predictive screening of therapeutic agents. Poly lactide co-glycolide (PLGA) polymer is frequently used in the development of porous substrates for complex cell culture. Substrates or scaffolds with highly interconnected, micrometric pores have been shown to positively impact tissue model formation by enhancing cell attachment and infiltration. We report a novel alginate microsphere (AMS)-based controlled pore formation method for the development of porous, biodegradable PLGA microspheres (PPMS), for tissue engineered lung tumor model development. The AMS porogen, non-porous PLGA microspheres (PLGAMS) and PPMS had spherical morphology (mean diameters: 10.3 ± 4, 79 ± 21.8, and 103 ± 30 μm, respectively). The PPMS had relatively uniform pores and a porosity of 45.5%. Degradation studies show that PPMS effectively maintained their structural integrity with time whereas PLGAMS showed shrunken morphology. The optimized cell seeding density on PPMS was 25 × 10^3^ cells/mg of particles/well. Collagen coating on PPMS significantly enhanced the attachment and proliferation of co-cultures of A549 lung adenocarcinoma and MRC-5 lung fibroblast cells. Preliminary proof-of-concept drug screening studies using mono- and combination anti-cancer therapies demonstrated that the tissue-engineered lung tumor model had a significantly higher resistance to the tested drugs than the monolayer co-cultures. These studies indicate that the PPMS with controllable pore diameters may be a suitable platform for the development of complex tumor cultures for early *in vitro* drug screening applications.

## Introduction

According to American Cancer Society estimates, lung cancer is the most commonly diagnosed human cancer, with 228,150 new cases (116,440 in men and 111,710 in women) and 142,670 deaths (76,650 in men and 66,020 in women) expected from this condition in 2019 (Siegel et al., 2019). Despite the advances in sophisticated early diagnostic techniques and treatments, the overall prognosis of lung cancer patients remains poor with a 5-year survival rate of only 15% (Edelman et al., 2001). Further, the development of an anti-cancer drug by pharmaceutical companies is exceedingly expensive with an estimated $648 million spent in this process; yet 95% of anti-cancer drugs in clinical trials tend to fail due to toxicity and inefficacy despite demonstrating acceptable anti-cancer activities during preclinical experiments (Moreno and Pearson, 2013; Prasad and Mailankody, 2017). The differences in the outcomes can be partly attributed to the fact that currently available *in vitro* cancer models fail to recapitulate clinical cancer conditions, and hence often provide inaccurate results during drug development. Two-dimensional (2D) culture of cells as a monolayer on glass or tissue culture plastic (TCP) are most commonly used for *in vitro* investigation of drugs, but these fail to mimic the three-dimensional (3D) nature of *in vivo* environments. While *in vivo* models can give more reliable results, it is not feasible for large-scale drug screening purposes at preliminary stages of drug development. A major barrier to cancer drug discovery today, therefore, is the lack of predictive experimental *in vitro* human tumor models for early screening of promising drug candidates.

There has been a paradigm shift especially in the last decade toward the development of 3D *in vitro* tumor models that can recapitulate the tumor microenvironment for reliable chemotherapeutic drug testing and optimization. Three-dimensional models proposed include spheroids developed using spinner flasks (McMillan et al., 2016; Yakavets et al., 2017), non-adherent dishes or hanging drop method (Costa et al., 2018), scaffolds (McMaster et al., 2019), gels made of extracellular matrix components like collagen (Liu C. et al., 2018), and microparticles mostly of non-uniform porosities and pore-sizes (Sahoo et al., 2005). However, several models being studied today have faced issues in terms of maintaining reproducibility between batches, allowing uniform diffusion of oxygen and nutrients to enable cell growth, and controlling the sizes of the tissue models formed (Choi et al., 2010). For example hanging drop method is limited by tedious steps and the difficulty in changing media, spinner flask method involves long-term incubation for the development of spheroids, and non adherent 3D culture approaches often require an extra step of coating the surface with non-adherent material, which can lead to higher costs or uneven coating (Wang and Yang, 2008; Patel et al., 2015). Microspheres offer greater advantage over other methods as it provides a large surface area for cell attachment and proliferation (Hacker et al., 2003). Porous microspheres facilitate attachment, proliferation, infiltration, and extracellular matrix production by the cells (Horning et al., 2008). Besides, porous microspheres offers better control over the spatial and physical parameters of the *in vitro* tumor models formed, compared to other approaches (Horning et al., 2008). This will reduce batch-to-batch variations and help obtain consistent and repeatable results during *in vitro* pharmaceutical drug testing. Both large polymeric porous (PPMS) and non-porous (PLGAMS) microspheres prepared using biocompatible, biodegradable polymers like PLGA (poly lactic-co-glycolic acid) have been used previously as substrates for development of tumor and tissue models *in vitro* (Sahoo et al., 2005; Horning et al., 2008; Kang et al., 2008; Kang and Bae, 2009). We have previously reported the development of PPMS using different kinds of porogens for lung tumor model development (Kuriakose et al., 2019). The pores on these particles, were non-uniform and too small to allow cell infiltration.

**FIGURE 1:**
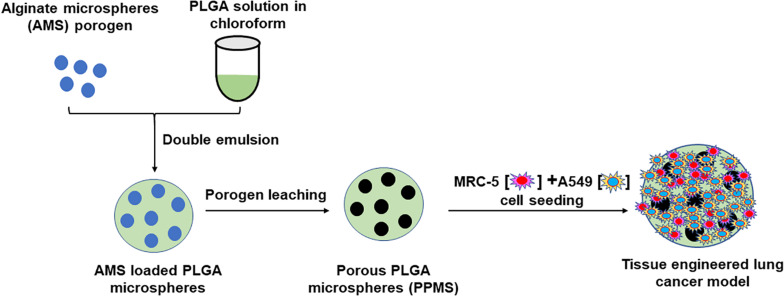
Schematic representation of preparation of Porous PLGA microspheres using AMS as porogen and its use in the development of lung tumor model.

Based on the above background, we hypothesized that large PLGA microparticles made porous using our innovative controlled pore formation method would facilitate the generation of a more representative lung tumor model than other techniques. Our rationale was that the interconnected pores of controlled diameters on the PLGA microparticles will facilitate uniform nutrient, oxygen, and waste diffusion, which in turn will strongly influence uniform cell distribution throughout the scaffold, and cell infiltration and growth through the pores. Our novel alginate microsphere (AMS) porogen-based controlled pore formation method is an improvement over existing approaches for making scaffolds with large, relatively uniform, interconnected pores for tissue engineering applications. A schematic representation of the preparation of porous PLGA microspheres using AMS as porogen, and its use in the development of lung tumor model is shown in Figure 1. Developed PPMS were characterized by advanced analytical techniques. We report for the first time the generation of lung cancer co-cultures using A549 lung adenocarcinoma cells and MRC-5 human lung fibroblasts, on PPMS. A comparative correlation between PPMS and PLGAMS was also generated. Further, series of anticancer drugs were tested alone and in combination using the fabricated *in vitro* lung cancer model and compared with 2D co-culture system. To determine the drug efficacy in 2D monolayer and developed lung cancer tumor model, we tested six anti-cancer drugs Cisplatin (Cis), Doxorubicin (Dox), Curcumin (Cur), Paclitaxel (Ptx), Etoposide (Eto), Gemcitabine (Gem) and the combinations of Cis with Eto or Gem, which are currently used in clinical practice for the treatment of lung cancer. Cell viability and confocal imaging was performed after drug treatment to assess the responses of the 2D and the PPMS-based models to the same concentration of drugs.

## Materials and Methods

### Formulation and Characterization of AMS Porogen

The AMS porogen was formulated by water-in-oil emulsion technique as described by Zhu et al. (2005) with minor modifications. Briefly, 5 ml of aqueous phase [3.1% w/v sodium alginate solution in de-ionized (DI) water] was emulsified in 14 ml of oil phase (1.97% v/v Span 85 solution in soybean oil) by vortexing for 10 min. Then, 1 ml of 16.4% v/v tween 80 solution in soybean oil was added and vortexed again for 10 min. AMS formed in the emulsion were crosslinked by dropwise addition of 3.5 ml of CaCl_2_ solution (10% w/v) while stirring at 500 rpm, 20 min. AMS were hardened by dropwise addition of isopropyl alcohol (IPA) and then centrifuged at 200g for 10 min at RT (23°C). The particles were then washed with IPA: water (50:50% v/v) once and then with distilled water (DW) to ensure complete removal of oil and surfactants. Generated pellet was resuspended in DW and stored at 4°C for further characterization. Morphology and diameter of the AMS was analyzed by brightfield microscope (EVOS^®^ FL Auto Cell Imaging System, Life Technologies corporation, Bothell, WA, United States).

### Formulation of PPMS and PLGAMS

Non-porous PLGA microspheres were prepared by a standard single emulsion (oil-in-water) solvent evaporation technique (Chaisri et al., 2009) whereas PPMS was developed by double emulsion solvent evaporation technique using AMS as the porogen. For PLGAMS, the organic phase [10% w/v of PLGA (Sigma Aldrich, United States, Resomer RG 503H, acid terminated; lactide:glycolide ratio 50:50, Mol wt 24,000 to 38,000) in 1 ml of chloroform] was added dropwise to 20 ml solution of 0.5% w/v polyvinyl alcohol (PVA). The formulation was stirred overnight for solvent evaporation, and then centrifuged at 4000 rpm at 10°C for 10 min to yield purified PLGAMS. For PPMS, the primary emulsion was prepared by vortexing AMS (3, 6, or 9 mg) with 2 ml of organic phase (2.5% w/v of PLGA in chloroform). This primary emulsion was then added dropwise in 10 ml 0.5% w/v PVA solution and stirred overnight for solvent evaporation. The formulation was then centrifuged at 4000 rpm at 10°C for 15 min prior to treatment with 1 ml of EDTA solution (14.62 mg/ml) to digest AMS. The mixture was then again washed with DW to yield purified PPMS. Both the particles were then finally freeze dried and stored at −20°C for further characterization.

### Characterization of PLGAMS and PPMS

Formulated PLGAMS, and PPMS were evaluated for particle size and morphology using EVOS^®^ FL Auto Cell Imaging System and Scanning electron microscope (SEM, Zeiss Sigma, United States). Images of all formulations were analyzed for particles size distribution by ImageJ software. Porosity of PPMS was analyzed with liquid displacement method following literature’s method (Nazarov et al., 2004; Zu et al., 2012). Energy-dispersive X-ray spectroscopy (EDS) was performed to confirm porogen leaching. Both PLGAMS and PPMS were also evaluated using cryo-SEM to confirm the internal structure of particles. For cryo-SEM, a small drop of the sample (approximately 5 μl) was placed on the holder and frozen in liquid nitrogen slush. Frozen specimen was immediately placed in the vacuum chamber of SEM and cooled to −130°C where the particles were fractured using a cold flat-edge knife and imaged using FE-SEM system.

### Degradation Studies on PLGAMS and PPMS

Particles were sterilized by treatment with 70% ethanol, before use in the experiment. To study the degradation kinetics, 3 mg of either PPMS or PLGAMS in pre-weighed tubes was incubated in 2 ml of Dulbecco’s Modified Eagle Medium (DMEM) completed using 10% fetal bovine serum and 1% penicillin-streptomycin, at 37°C. At predetermined timepoints (day 1, 2, 4, 7, 14, 21, and 28), the particles were washed, centrifuged (2000 rpm, 10 min at 10°C) and lyophilized. Particle weights were measured at different time points and the surface morphology was determined by SEM.

### *In vitro* Cell Culture Experimentation

MRC-5 lung fibroblast (Catalog CCL-171) and A549 lung epithelial cells (Catalog CCL-185) were purchased from American Type Culture Collection (ATCC, Manassas, VA, United States), and cultured in complete DMEM. MRC-5 at passages 3–6 were used throughout our studies. For all *in vitro* experiments, agarose-coated 96 well plates were used. These were prepared by adding 50μl of 1% agarose to the wells followed by sterilization under UV light. The particles used in the experiments were sterilized by 70% ethanol.

### Degradation Behavior of PLGAMS and PPMS in Presence of A549 Lung Cancer Cells

To visualize particle degradation in the presence of cells, cells at seeding density of 5 × 10^4^ cells/well were added to agarose-coated wells containing sterilized PPMS or PLGAMS. The plates were kept in a CO_2_ incubator at 37°C and the media was replaced every 3 days. At predetermined time points (1, 2, 4, 7, 14, 21, and 28 days), half of the particles were trypsinized whereas the other half was fixed by using 2.5% glutaraldehyde for 20 min, followed by treatment with 50, 60, 70, 80, 90, and 100% v/v ethanol for 10 min each. The two halves were dried in hood for solvent evaporation and visualized by SEM.

### Optimization of Cell Seeding Density

For this study, A549 cells at different seeding densities (5 × 10^3^, 10 × 10^3^, 25 × 10^3^, and 50 × 10^3^ cells/well/mg of particles) were incubated with sterilized PPMS or PLGAMS on agarose-coated well plates. The media was replaced every 3 days. At predetermined time points (1, 3, 5, 7, 14, and 21 days), the media was carefully removed from each well using a 26G syringe and WST-1 assays were performed as per manufacturer’s instructions. A cell standard curve was plotted from absorbance values in WST-1 assays of known number of cells (5 × 10^3^, 10 × 10^3^, 25 × 10^3^, 50 × 10^3^, and 100 × 10^3^ cells/well). Based on the calibration curve obtained, the number of viable cells on the PPMS and PLGAMS at each timepoint was determined. Further Live/Dead^TM^ assay (Invitrogen, Carlsbad, CA, United States) was done as per the standard protocol on days 7 and 14 to confirm cell viability. Stained cells on particles were observed under Confocal laser scanning microscope (CLSM, Nikon Eclipse Ti2) with bright field, FITC (green), Cy5 (red) channels.

### Coating of Collagen on PPMS

Collagen is known to improve the cell attachment and allow better proliferation on the substrate (Kleinman et al., 1981). The PPMS were coated with collagen type I (C7661, Sigma-Aldrich, United States) as per the method by Hong et al. (2005) with slight modifications. Briefly, PPMS were immersed in 6% v/v of hexanediamine in n-propanol solution with stirring for 8–10 min at room temperature. After 10 min, the particles were washed thoroughly using DW. The surface modified PPMS were then immersed in 1% glutaraldehyde solution at room temperature for 4 h and then thoroughly washed using DW to ensure complete removal of excess glutaraldehyde. The PPMS were then immersed in a solution of 0.5% collagen in 3% acetic acid at 4°C for 24 h with occasional shaking. Collagen coated PPMS (Col-PPMS) were washed with DW to remove the excess of collagen, and then lyophilized for further characterization.

### Characterization of Collagen-Coated PPMS

Mouse Collagen I Monoclonal Antibody (catalog CSI 008-01-02) and goat anti-mouse IgG-FITC (catalog sc-2010) antibodies were purchased from Invitrogen and Santa Cruz Biotechnology, respectively, and were used without further purification. Lyophilized PPMS and Col-PPMS were first dissolved in PBS and blocked with 5% bovine serum albumin (BSA) for 1 h at 4°C. Then the particles were washed with Tris buffer saline with 0.1% v/v tween-20 (TBST) before incubation with Collagen I monoclonal antibody (dilution 1:1000) overnight at 4°C. Next, the particles were washed with TBST 3–4 times and incubated with secondary antibody (dilution 1:2500) for 4 h followed by washing with TBST. At each step, particles were collected via centrifugation at 100 *g* in for 5 min. Finally, Col-PPMS and PPMS were observed under Nikon CLSM with bright field and FITC channels.

### Co-culture of A549 and MRC-5 on PPMS

Studies were done to ensure that both A549 and MRC5 cells can attach and grow on the particles, and that both cells are viable. One milligram of either Col-PPMS or PPMS suspension in DMEM were added to the agarose pre-coated 96-well plates prior to cells seeding. Then, MRC-5 and A549 cells were separately pre-stained with Vybrant DiO and DiD cell-labeling solutions (Invitrogen), respectively followed manufacture’s protocol, before addition onto the particles. The pre-stained cells were mixed in DMEM at 1:1 cell ratios and then seeded at 25 × 10^3^ cells/mg/well on either Col-PPMS or PPMS suspension. At each time point, particle with cells were collected, washed with Dulbecco’s phosphate buffered saline (DPBS) and imaged using Nikon Inverted Confocal Microscope with FITC and Cy5 channels for MRC-5 and A549 cells, respectively. In parallel, non-stained MRC-5 and A549 were seeded at 25 × 10^3^ cells/mg of PPMS or Col-PPMS/well (1:1 ratio of cells). At each time point (day 1, 3, 5, and 7), the media was removed, the particles were washed with DPBS, and WST-1 assays were performed. Separately, cells on the particles were stained with Live/Dead^TM^, and then observed via Nikon CLSM with FITC and Cy5 channels for live and dead cells, respectively.

### Screening of Anti-cancer Drugs Using Tissue Engineered Lung Tumor Model and 2D Models

The anti-cancer effect of drugs was studied by using the developed co-culture lung tumor model and its corresponding 2D cell culture. The optimized seeding density of 25 × 10^3^ cells/mg/well was used to seed the cells at 1:1 ratio on Col-PPMS in the agarose coated 96 well plate. Cells were incubated for 5 days to develop the lung tumor model on PPMS. The average number of cells growing on PPMS at day 5 was determined to be 22 × 10^3^ cells/well based on our cell seeding optimization studies. Therefore cells were seeded at 22 × 10^3^ cells/well for the monolayer culture, 1 day before the start of anti-cancer screening. The protocol for cell seeding was optimized to obtain approximately same cell count in both experimental groups. The PPMS-based lung tumor model and the 2D cell cultures were starved for 6 h in serum free media and then exposed to anti-cancer drugs at IC_50_ values such as Cis (24.27 μM) (Zhuo et al., 2015), Dox (1.4 μg/ml) (Hu et al., 2008), Cur (36.69 μg/ml) (Zhu et al., 2017), Ptx (2.36 μg/ml) (Yuan et al., 2008), Eto (44 μM) (Rho et al., 2009), and Gem (47.7 μM) (Rho et al., 2009). To evaluate the effect of Cis in combination with Eto and Gem, two strategies were used. In the first strategy, the cells was exposed to separately to either of the two drug combinations for 24 and 72 h (simultaneous addition). In the second strategy, the cell cultures were exposed to Cis for 24 h and then with Eto or gemcitabine for another 48 h (subsequent addition). All the samples were evaluated after 24 and 72 h using WST-1 assay and Live/Dead assay as per the standard protocol.

## Results

### Formulation and Characterization of Porogen

**FIGURE 2:**
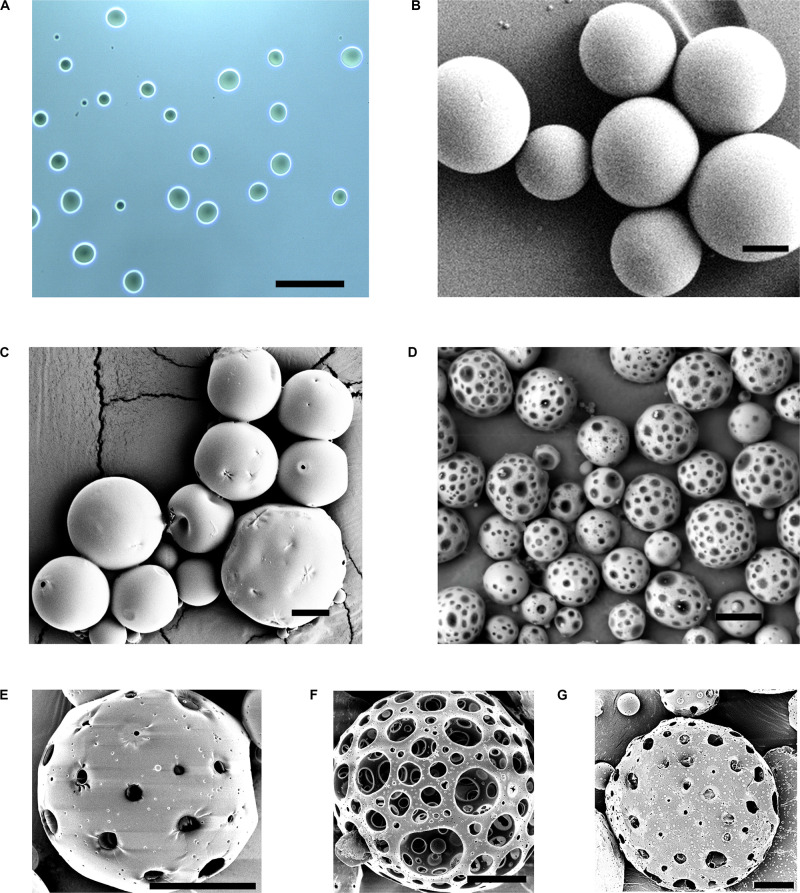
Characterization and optimization of PPMS. Brightfield image of **(A)** AMS porogens showing uniform spherical morphology. SEM images of **(B)** PLGAMS showing smooth, spherical morphology, **(C)** PPMS before porogen leaching with rough surfaces, and **(D)** PPMS after porogen leaching with relatively uniform pores on the structure upon removal of AMS; SEM images of optimization of PPMS based on ratio of AMS (mg) to PLGA (mg) (AMS:PLGA) used in the formulation – **(E)** 3:50, **(F)** 6:50, and **(G)** 9:50; Scale bars: **(A,B)**, 100 μm; **(C)**, 20 μm; **(D)**, 50 μm; **(E–G)**, 20 μm.

Alginate microsphere were successfully formulated by emulsion method and characterized by brightfield microscope to evaluate the particle diameter and morphology. They had a mean diameter of 10.3 ± 4 μm, and spherical morphology (Figure 2A). A 1:2 v/v ratio for tween 80: span 85 and 10% w/v aqueous solution of calcium chloride were found to be optimal for obtaining AMS with uniform particle sizes. EDS analysis of AMS shows the presence of Ca ion peak confirming the crosslinking (Supplementary Figure S1).

### Formulation and Characterization of PLGAMS and PPMS

**FIGURE 3:**
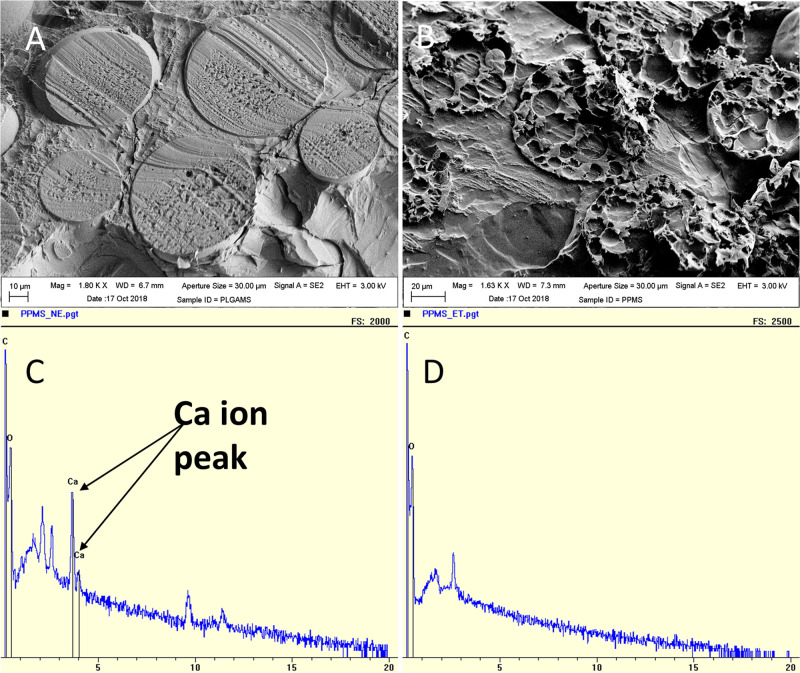
Characterization of pores on PPMS. Cryo-SEM images of **(A)** PLGAMS (scale bar: 10 μm) showing smooth surface; **(B)** PPMS (scale bar: 20 μm) with uniform interconnected porous structure. EDS analysis of **(C)** PPMS before leaching by EDTA showing calcium peaks due to presence of calcium crosslinked AMS within the particles; and **(D)** disappearance of calcium peak in the PPMS after leaching of AMS porogens by EDTA treatment.

The PLGAMS had a smooth surface with clear spherical morphology and a mean diameter of 79 ± 21.8 μm (Figure 2B). PPMS also showed predominantly spherical morphology with diameter of 103 ± 30 μm. Controlled pore formation on the PPMS was achieved by digesting the incorporated AMS. The surface of PPMS pre-AMS digestion was rough (Figure 2C), while it became porous (Figure 2D) post digestion of AMS using EDTA. Using ImageJ with particle analysis tool (Supplementary Figure S2), the pore diameter was determined as 7.4 ± 4.4 μm. Using liquid displacement method (Nazarov et al., 2004; Zu et al., 2012), porosity of PPMS was determined as 45.5%. SEM images of different formulations (Figures 2E–G) clearly shows that the 6:50 w/w ratio of AMS: PLGA generated PPMS with well-distributed, interconnected pores, and this formulation was chosen for further investigation. Internal structure of PLGAMS and PPMS analyzed by cryo-SEM (Figures 3A,B) clearly demonstrated the uniform, interconnected pore network within the PPMS whereas PLGAMS showed solid and impermeable structure. EDS spectra of PPMS without EDTA treatment showed the presence of prominent Ca^++^ ions peaks whereas the EDTA treated PPMS did not show the presence of Ca^++^ peak which confirms the efficient digestion of AMS (Figures 3C,D).

### Degradation Studies on PLGAMS and PPMS

**FIGURE 4:**
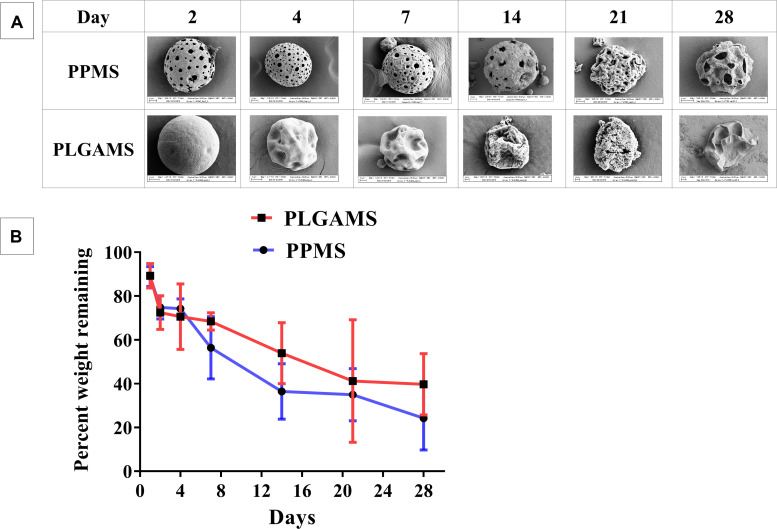
Degradation studies. **(A)** SEM images of PPMS and PLGAMS in complete media at 37°C PPMS shows degradation with clear pores until 28 days whereas PLGAMS degradation results in distorted morphology of the particles due to autocatalytic effect; **(B)** decrease in weights of PPMS and PLGAMS in cell media at 37°C observed over time.

The effect of complete media on PLGAMS and PPMS in terms of morphology and changes in sample weight were investigated. SEM images (Figure 4A) taken at different timepoints clearly show that the PPMS effectively maintained its structural integrity with time whereas PLGAMS showed shrunken morphology. Both PLGMS and PPMS underwent significant changes in weight within 3 weeks (Figure 4B). However, the differences in weight between both particles were not statistically significant. A greater loss in weight of PPMS (75.8 ± 14.5% of original weight) was observed on day 28 when compared to PLGAMS (60.3 ± 14% of original weight).

### Degradation Behavior of PLGAMS and PPMS in Presence of Cells

The effect of A549 cell growth on the particles was studied. Images from SEM clearly demonstrate that both PPMS and PLGAMS supported cell growth. After trypsinization, the cells were successfully detached from the particle surface and the pores were clearly visible in PPMS, whereas in PLGAMS the surface morphology was distorted with time (Supplementary Figure S3). Images also reveals that the pore structure in PPMS was intact for 28 days.

### Optimization of Cell Seeding Density

**FIGURE 5:**
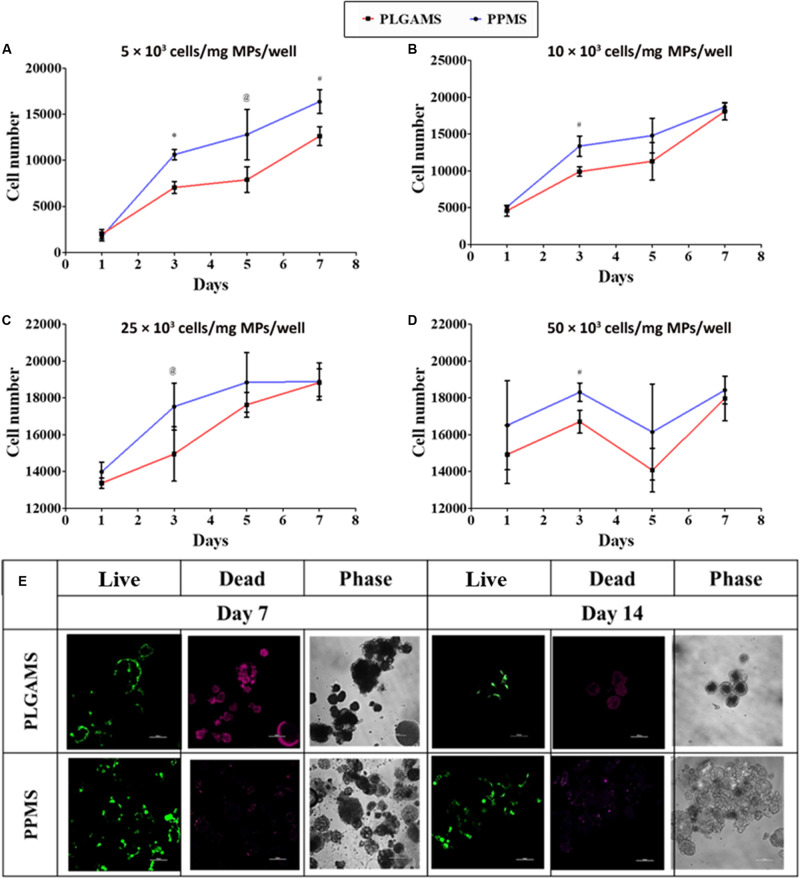
Cell seeding optimization experiments. PPMS facilitated greater A549 cell attachment and growth compared to PLGAMS. Seeding densities used include **(A)** 5 × 10^3^, **(B)** 10 × 10^3^, **(C)** 25 × 10^3^, **(D)** 50 × 10^3^ cells/mg of microparticles/well. Data as mean ± SD, *n* = 3; **(E)** Live/Dead staining shows greater cell death (red) on PLGAMS than on PPMS, which had more viable cells (green) (optimized cell seeding density – 25 × 10^3^ cells/mg/well; scale bars = 100 μm). Significant difference was analyzed by Student’s *t*-test (*p < 0.001, ^#^*p* < 0.01, ^@^*p* < 0.05).

WST-1 assay was performed to determine the optimal seeding density for cell culture on PPMS and PLGAMS (Supplementary Figure S4). Cells growing on PPMS gave comparatively higher absorbance values than those growing on PLGAMS. There was a significant difference between cell attachment on PPMS and PLGAMS at 5000 (Figure 5A), 10,000 (Figure 5B), and 25,000 (Figure 5C) cells per well density, whereas 50,000 cells/well (Figure 5D) did not show a linear growth of cells. Based on the growth curves, 25,000 cells/well was considered to be optimal. Approximately 18,800 cells/mg of PPMS was observed on day 5 using this seeding density, compared to 17,625 cells/mg of PLGAMS on the same day. All the seeding densites except for 50,000 cell/well demonstrated the log phase till day 7 followed by a lag phase after day 7 time point. Live/Dead analysis of PPMS and PLGAMS on day 7 and 14 are shown in Figure 5E. PPMS shows more of live cells with very less dead cells when compared to PLGAMS on day 7 and 14.

### Collagen Coating on PPMS

**FIGURE 6:**
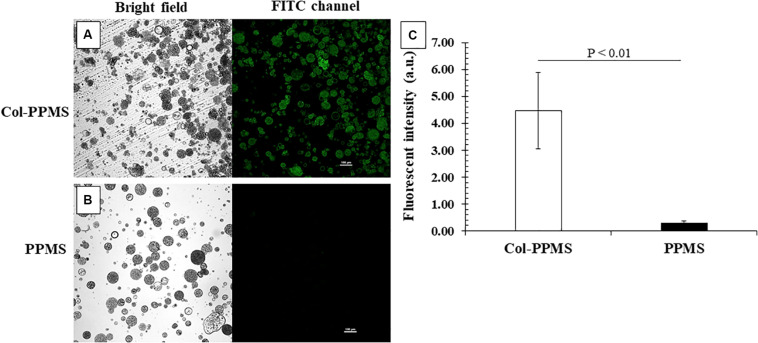
Confirmation of collagen coating on PPMS by immunostaining. Green fluorescent particles indicated successful collagen coating on **(A)** Col-PPMS in comparison to **(B)** non-coated PPMS. Scale bar: 100 μm. **(C)** Fluorescence intensity quantified by ImageJ showing significantly higher fluorescence from Col-PPMS than from non-coated PPMS. Significant difference was analyzed by Student’s *t-*test.

The qualitative and quantitative outcome of the collagen coating on PPMS was confirmed by immunochemistry. It can be observed from Figures 6A,B that col-PPMS strongly expressed green fluorescence whereas non-coated PPMS yielded none. The fluorescence was produced by the FITC conjugated secondary antibodies where they bound to the anti-collagen primary antibodies. Our images confirm qualitatively that the anti-collagen antibodies could successfully bind to the collagen coating on Col-PPMS. Analyzing fluorescent intensity with ImageJ also showed a significant difference (Figure 6C) between the two groups (*p* = 0.003 in *t-*test for two groups with two-tail distribution and unequal variance), confirming that the collagen coating on PPMS was successful.

### Viability of A549-MRC-5 Co-cultures on the Microsphere Substrates

**FIGURE 7:**
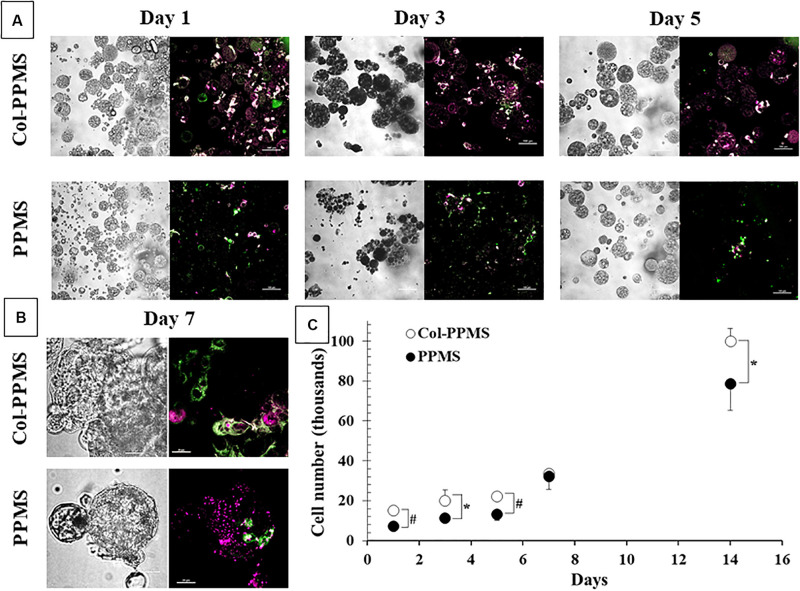
Co-culture of MRC-5 and A549 (1:1 seeding ratio) at **(A)** low magnification (scale bar: 100 μm) and **(B)** high magnification (scale bar: 20 μm). Left, phase contrast images; right, overlay of green (FITC channel) and red (Cy5 channel) fluorescent images. **(C)** Quantification of number of cells attached onto Col-PPMS and PPMS showing significantly higher cell attachment on Col-PPMS at almost all timepoints. *p < 0.05 and ^#^*p* < 0.01 with respect to cells growing on PPMS of the same day of study.

It can be observed from Figures 7A,B that from days 1 through 7, there were more cells bound to collagen-coated PPMS than those on non-coated PPMS. Confocal images from day 5 (low magnification) and day 7 (high magnification), shows an overlap of red and green fluorescence (light green to light yellow) which indicates that the MRC-5 (DiO dye, green) and A549 (DiD dye, red) can be co-cultured successfully on the same particles of collagen-coated PPMS. To study cell growth on PPMS and PLGAMS, WST-1 cell viability assays were used and absorbance readings from the cells grown on particles were compared to those from a cell standard. It can be observed from Figure 7C that there were more cells binding on collagen-coated PPMS. Significance difference was obtained for all single day except day 7 with *p* < 0.05 or *p* < 0.01 on *t-* tests. In accordance with WST-1 studies, Live/Dead assays also showed more alive cells (green fluorescence) and less dead cells (red fluorescence) on Col-PPMS compared to non-coated PPMS (Supplementary Figure S5).

### Screening of Anti-cancer Drugs Using the Tissue Engineered Lung Tumor and 2D Models

**FIGURE 8:**
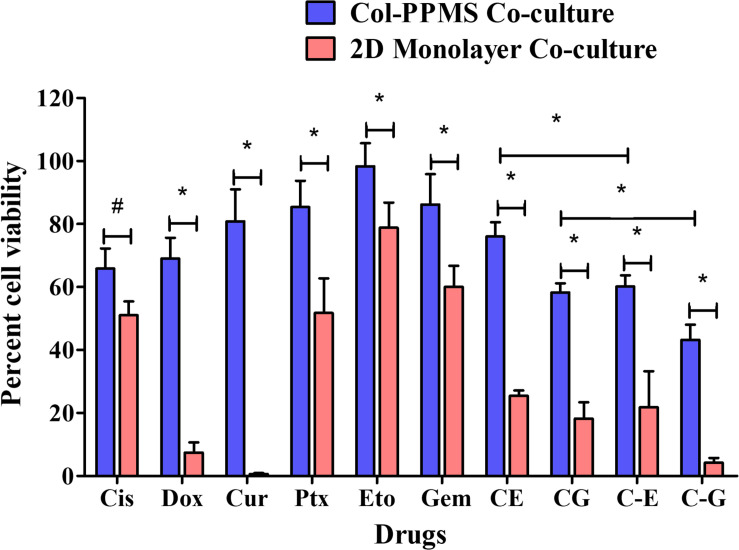
*In vitro* drug screening studies. Comparative *in vitro* screening of anti-cancer drugs on A549-MRC-5 monolayer and col-PPMS co-culture treatment groups after 72 h of treatment showed significant difference in cell viability between both cultures. The col-PPMS-based cultures showing significant higher resistance to the drugs than monolayer cultures. Significance was analyzed by Student’s *t-*test (*p < 0.001, ^#^*p* < 0.01).

**FIGURE 9:**
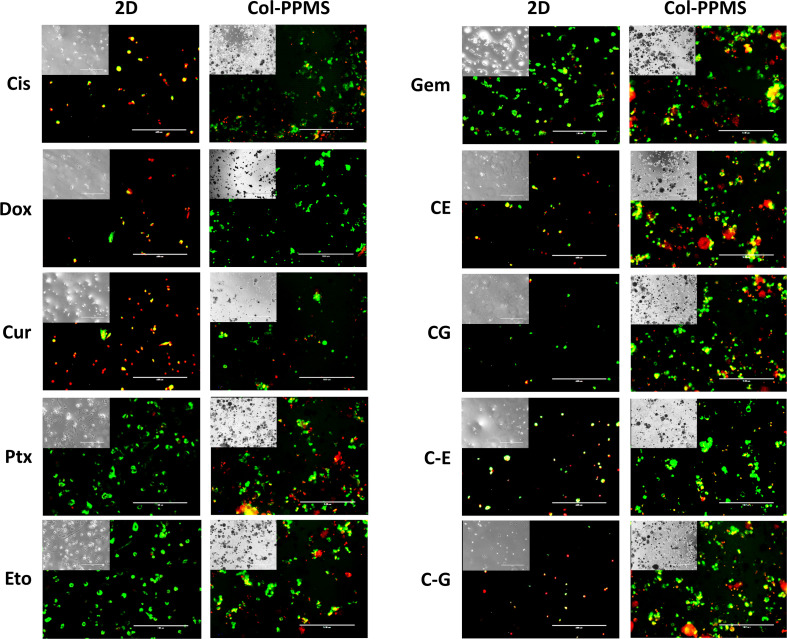
Fluorescence Live/Dead imaging of monolayered and col-PPMS-cocultured A549 and MRC-5 after 72 h of drug treatment showing greater viability (green) for col-PPMS co-cultures than for the monolayer co-cultures in almost all cases. For the monolayer co-cultures, most cells were either washed away or floating due to cell death, following treatment. Insert images are phase contrast images of the same view. Scale bar: 400 μm.

Efficiency of anti-cancer drugs such as Cis, Dox, Cur, Ptx, Eto, Gem, and the combinations of Cis with Eto or Gem were evaluated using the developed *in vitro* lung tumor model and the 2D monolayer co-culture after 24 (Supplementary Figure S6) and 72 h (Figure 8). After 24 h of treatment with the anti-cancer drugs, a significant difference was noticed between the Col-PPMS cultures and 2D monolayer co-cultures in their responses to Cur, Cis + Eto (CE), and Cis + Gem (CG) combinations. However, after 72 h of treatment, a significant difference in the responses of the Col-PPMS cultures to all the drugs was observed, when compared to the 2D monolayers. In combination treatment, it was observed that successive addition of drugs (C−E and C−G) respectively resulted in greater cell death when compared to simultaneous addition (CE and CG). All drugs followed the same trends for both models – greater viability was observed for cells growing on Col-PPMS compared to those growing as a monolayer, when subjected to the same concentration of drugs. Specifically, successive addition of Cis and Gem demonstrated most anti-cancer activity while Eto was the least toxic toward cells cultured on Col-PPMS. Cis and Gem combination also demonstrated better efficacy compared to the combination of Cis and Eto when tested against the A549-MRC-5 co-cultures on Col-PPMS. The Cis – Eto combination did not show significant difference in 2D monolayer co-culture in both the successive and subsequent treatment strategies, whereas a significant difference was observed in the response of the Col-PPMS co-cultures to both treatments. The Col-PPMS model also demonstrated significant resistance to the combination of Cis – Gem than the 2D monolayer cultures for both treatment strategies. Live/Dead images of co-cultured cells at 6 and 24 h (Supplementary Figures S7–S10) post treatment showed clear anti-cancer effects of the drugs for both monolayer and Col-PPMS cultures (Supplementary Figures S7, S10). Treatment of the models with drugs for 72 h (Figure 9) resulted in greater cell death in both models, which concurs with the data obtained.

## Discussion

Monolayer cell culture models are extensively used to screen and investigate the efficacy of anti-cancer drugs. However, they are unable to mimic cell-cell interactions, paracrine signaling and cell-matrix interactions seen in an *in vivo* environment (Kapałczyńska et al., 2018). Although epithelial cell properties are mimicked reasonably well in both 2D and 3D (Duval et al., 2017), the differences for cancer cells (and stem cells) are significant, especially in term of gene expression (Loessner et al., 2010), cell proliferation for tumor development (Duval et al., 2017), migration for cancer metastasis (Tibbitt and Anseth, 2009; Duval et al., 2017) and stiffness for drug resistant (Duval et al., 2017). The 3D cell cultures are necessary to represent drug, oxygen and nutrient diffusions, as well as cell necrotic/proliferative states that reflect more accurately the characteristics of the *in vivo* environment (Kapałczyńska et al., 2016; Duval et al., 2017). Further, cells in 2D culture are confined to a single plane and thus encounter minimal or no resistance from the surrounding ECM. In this manner, they demonstrate higher migration rate compared to cells grown in tissue engineered scaffold models (Tibbitt and Anseth, 2009). Therefore there is a significant difference in the expression of specific biomarkers, generation of extracellular matrix, metabolic function, and cellular receptors from the scaffold-based tissue culture models to 2D (Horning et al., 2008).

While several techniques for culturing cells in a 3D format have been developed in the past decade, most techniques are limited by batch-to-batch variability, cumbersome techniques, high cost, and low throughput methods (McMillan et al., 2016; Yakavets et al., 2017; Costa et al., 2018). In this work, we propose the use of AMS as a novel porogen which allows us to control pore formation on polymeric substrates, which can then be used to develop tissue engineered lung tumor models. AMS formulated by water-in-oil emulsion technique showed particle diameter in the range of 10.3 ± 4 μm. Zhu et al. (2005) had used a similar method for the synthesis of AMS for drug delivery application, and their particles had an average diameter of 4.5 μm and were uniformly dispersed as observed by us in this research. Unlike AMS, other porogens reported in the literature such as water, salt, ammonium bicarbonate and gelatin fail to produce uniform and large pores (Fan et al., 2013). The AMS-incorporated particles developed by us were treated with EDTA. The subsequent chelation with calcium ions led to reversing of the gel structure of AMS to fluidic state leading to the generation of relatively uniform pores (Chueh et al., 2010). Further, alginate, which remains stable in acidic media tends to degrade in alkaline medium by swelling followed by disintegration (Dhamecha et al., 2019). The calcium ions released by the ion exchange with sodium ions (alkaline) in the medium, and electrostatic repulsion between the carboxylate anions further accelerates the swelling and erosion of alginate gels (Lee et al., 2003) consequently leading to the generation of pores. EDS analysis confirmed that the peak of Ca^++^ disappeared upon treatment of PPMS with EDTA. The pore diameters on the PPMS were similar to the diameter of the entrapped AMS. Cryo-SEM images of particle cross-sections clearly demonstrated uniform and interconnected pore networks within the PPMS for cell and nutrient infiltration (Horning et al., 2008; Fan et al., 2013), whereas PLGAMS showed a solid and non-porous structure. Scaffolds with interconnected pores made using synthetic biodegradable polymers, have been extensively used in tissue engineering for cell growth and differentiation (Zhang et al., 2015; Kuriakose et al., 2019).

The key factor in selection of polymer for the development of porous scaffold is its biocompatibility, biodegradability, degradation behavior and mechanical strength of developed scaffold system. Once exposed to media and cells, a porous scaffold should maintain mechanical properties and structural integrity until the loaded cells adapt to the environment and excrete enough extracellular matrix, etc. The degradation rate of porous scaffolds affects cell vitality and growth overtime. Based on the above the requirements, we selected PLGA (Mol wt 24,000–38,000) which have all the desired properties. PLGA is a well-known USFDA approved biocompatible and biodegradable polymer used for drug delivery. The polymer is robust and shown to be used in tissue engineering. It degrades through chemical hydrolysis of the unstable ester bonds to form lactic acid and glycolic acid as non toxic byproducts. Further for tissue engineering applications, there is a need of desirable mechanical strength which was provided by PPMS (Wu and Ding, 2004). For the development of PPMS based lung tumor model, there is a requirement of sufficient mechanical strength so that the scaffold can withstand the experimental condition for certain number of days until the tissue model is developed. PPMS have successfully used as a scaffold for various tissue engineering applications. A correlation of degradation in media and with cells overtime with changes in the morphology is explained in following sections.

In addition to pore interconnectivity, scaffolds must also have optimal degradation profiles to ensure that a stable substrate is available for the lung tumor model development (Gu et al., 2016). Both PPMS and PLGAMS underwent degradation in a linear fashion with time, with no significant difference. Nevertheless, SEM images clearly shows that the PPMS effectively maintained its structural integrity until day 28 while PLGAMS started to collapse after 2 weeks. The maintenance of structural integrity of PPMS is suitable for long-term culture of lung tumors. *In vivo* lung tumor models (injection of cancerous cell lines to nude mice) are generally ready after 1–2 week(s) (Onn et al., 2003) and last for 4–6 weeks (Onn et al., 2003; Isobe et al., 2013) when tumors reach a threshold size such as 4 mm diameter (Isobe et al., 2013). The reason for this distorted shape of PLGAMS can be explained by the autocatalysis process, where in the presence of media, ester linkages of PLGA particles undergo hydrolysis initiating the degradation process. In the non-porous particles, the acidic degradation products remains encapsulated within and accelerates the reaction. The eventual release of these highly concentrated degradation products upon complete polymer degradation will affect cell viability. On the other hand, the pores in PPMS provide channels for the catalytic degradation products to seep out thus resulting in comparatively intact morphology (Lu et al., 2000; Siepmann et al., 2005; Wu and Ding, 2005; Yang et al., 2008; Pan and Ding, 2012; Kuriakose et al., 2019). The greater decrease in weight of PPMS with time in Figure 4B could be explained by the removal of degradation byproducts during the washing step at each timepoint. On the other hand, the majority of the degradation byproducts of PLGAMS remained within the particles for the duration of the study, and were not cleared away during washing. The PPMS also maintained its structure following cell growth while PLGAMS became distorted following cell attachment, as observed during the degradation study.

Following particle characterization, studies done to optimize the cell seeding density for lung tumor model formation showed that a density of 25 × 10^3^ cells/mg/well was optimal. Greater cell growth was seen on PPMS both using WST-1 assays and Live/Dead analysis up to day 7. Similar saturation was also obtained after 6 days by Horning et al. (2008), who used porous poly lactic acid microspheres of 160 μm with ∼16 μm pore diameter as the scaffold for the development of breast cancer model seeding with MCF7.

Collagen, a key component of ECM, maintains the structural integrity (Yamada and Cukierman, 2007), facilitates cell attachment and proliferation (Horning et al., 2008), and is required for the growth of cells (Gillette et al., 2008). While greater cell attachment was observed on PPMS than PLGAMS, a collagen coating on PPMS further enhanced cell attachment and proliferation. Immunochemistry confirmed the successful coating of collagen on PPMS. The possibility of non-specific binding of primary antibodies was eliminated by the blocking process with 5% BSA solution prior to incubation with primary anti-collagen monoclonal antibodies followed by 4–5 washing cycles that eliminated any absorption on sphere surfaces.

Following collagen coating and optimization of seeding density, the Col-PPMS were used for co-culturing A549 and MRC-5 cells. It has been reported that the interplay of malignant and non-malignant cells in the tumor microenvironment has a major effect on cancer dynamics. In particular, fibroblasts have been reported to stimulate invasive behavior and metastasic activities of A549 both on 2D and on collagen-coated matrices (Ryszawy et al., 2018; Miyazaki et al., 2019). A549 also exhibited greater drug resistance with more DNA repair potential in the presence of WI-38 fibroblasts (Kobayashi et al., 2017). Furthermore, growth rate of A549 was also reported to be higher in MRC-5 co-culture than A549 alone (Chen et al., 2018). Therefore, to aim for an *in vitro* lung tumor model which closely mimics *in vivo* systems, we incorporated A549 lung adenocarcinoma cells co-cultured with MRC-5 lung fibroblasts on the collagen-coated porous particles (representing ECM). To the best of our knowledge, there is no reports of equivalent *in vitro* systems used for drug testing. Significantly higher A549 and MRC-5 attachment and growth was observed on the Col-PPMS than on PPMS. MRC-5 only favored Col-PPMS rather than non-coated PPMS; meanwhile, A549 were able to grow on both scaffold and at higher rate on Col-PPMS. The collagen coating may have facilitated better fibroblast growth (Miyazaki et al., 2019), while the aggressive nature of the cancerous cell line A549 may have allowed them to grow on both scaffolds. For skin tissue regeneration, Chen et al. have shown that the collagen coated micro sponges formed across the PLGA based mesh improved the proficiency of cell attachment and uniform proliferation of skin dermal fibroblasts (Chen et al., 2005). Similarly, Truong et al. (2012) demonstrated the use of different types and arrangement of collagen to improve the cell attachment of mouse fibroblasts on PLGA based fibers. Fibroblasts showed elongated and flat growth on collagen coated materials (Truong et al., 2012). Live/Dead staining images also confirmed greater cell death on the PPMS particles than those of Col-PPMS. Significant difference in cell viability and proliferation was seen at almost every timepoint when comparing Col-PPMS and PPMS. On day 1, significantly higher (*p* < 0.01) cell attachment was seen on Col-PPMS than on PPMS. A pair t-test with two-tailed distribution yielded a significant difference with *p*-value = 0.04, and this confirmed our hypothesis that overall MRC-5 and A549 grow more on collagen-coated PPMS than those on non-coated PPMS. Recently, there has been an alternative method wherein cell-derived decellularized matrix (DCM) produced by human fibroblasts improves cellular differentiation and function with more realistic *in vivo* micking model (Satyam et al., 2020). We propose to use this concept as the replacement of collagen in our future experiments.

Next, anti-cancer screening was performed on the developed *in vitro* lung cancer tumor model and 2D monolayer system. To the best of our knowledge, this is the first report on a lung cancer co-culture model developed using porous PLGA microspheres and evaluated for drug screening. Cis and Gem, which are used as the first line of chemotherapy for non-small cell lung cancer, had greater anti-cancer effects on cells cultured both as a monolayer and on the PPMS. The Col-PPMS co-cultures showed a significantly higher resistance to all of the tested drugs when compared to the 2D monolayer co-culture. Godugu et al. (2013) created alginate matrix for cancer spheroid development and the authors found out that IC_50_ of Docetaxel, Cis, Gem, and 5-fluorouracil against A549 in 3D were about 60-, 18-, 34-, and 30-fold, respectively, higher than those in 2D. They also observed the same resistance in 3D for H460 cell line (Godugu et al., 2013). The tendency that cancer cells become more robust in 3D models has not only been observed in collagen-coated systems, but also in scaffolds with other coatings. Collagen type I-coated and/or fibronectin-coated surfaces increased adhesion and proliferation of cancer cells grown both as monolayers (Zhang et al., 2013) and on porous polycaprolactone scaffold (Liu M. et al., 2018; Nayak et al., 2019). Yip and Cho observed significant Dox resistance of HepG2 human hepatocellular liver carcinoma spheroids cultured in collagen gel (∼100% viability) compared to spheroids not cultured in collagen gel (∼20% viability) (Yip and Cho, 2013) while Liu M. et al. (2018) observed that human ovarian cancer cell line OV-2008 became much more resistant to carboplatin, 5-fluorouracil and Ptx when cultured in a collagen matrix compared to cells cultured in the absence of collagen. Similarly, in our previous screening study using A549 cells cultured on fibronectin-coated porous PLGA microparticles, a two to fourfold higher drug resistance to Cis, Ptx, Dox, Gem, Cur, and 5-fluorouracil was observed when compared to 2D cultures (Kuriakose et al., 2019). This trend has been observed not just for lung cancer cultures but also for other cancer 3D models. For instance, MCF-7 cells cultured on porous chitosan coated PLA microspheres by Horning et al. (2008) were ∼21 and ∼12-folds more resistant to Dox and Ptx, respectively, than 2D cultures. Bulysheva et al. (2013) reported that HN12 oral and pharyngeal cancer cell lines grown on electrospun 3D scaffold were almost completely resisted to 40 nM of Ptx with almost 90% viability whereas the cells in 2D were very vulnerable with less than 5% viability.

The greater cell-cell interaction within our model, and the blocking of drug diffusion to the core due to the compact arrangement of cells (Millerot-Serrurot et al., 2010; Yip and Cho, 2013), would represent treatments given *in vivo* the co-culture with fibroblasts can also lead to increased drug resistance when compared to the monoculture model (Yip and Cho, 2013). It is reported that cancer associated fibroblasts can release TGF-β (growth factor) and help in cancer cell metastasis though increased generation of matrix metalloproteases. This in turn allows increased proliferation of cancer cells ultimately leading to increase in the resistance to chemotherapeutic agents (Wu et al., 2007; Yip and Cho, 2013). Fibroblasts boost the expression of soluble growth factors, cytokines and chemokines supporting the tumor cells and survival (Erdogan and Webb, 2017; Wang et al., 2017). This induces neovascularization and generation of immunosuppressive environment for enhanced tumor cell growth (Affo et al., 2017; Katayama et al., 2019; Monteran and Erez, 2019). Future studies will focus on mimicking the hypoxic core of tumors using our col-PPMS model, as well as incorporation of more cells and cues present in the tumor microenvironment. We will also further develop the model using human patient-derived cells to more closely resemble the *in vivo* conditions for predictive drug screening.

## Conclusion

We have successfully synthesized PPMS with large, interconnected pores. Although PPMS have been used in various research applications, we report an innovative method of safe and controlled pore formation using AMS and EDTA on PLGA microspheres. Controlled pore formation on the PPMS was achieved by digesting the incorporated AMS. Degradation studies of formulated PPMS and PLGAMS clearly show that the PPMS effectively maintained its structural integrity with time whereas PLGAMS showed shrunken morphology. Collagen coating of the particles demonstrated significant improvement in the attachment and proliferation of A549-MRC-5 co-cultures. To the authors’ knowledge, this is the first study to use PPMS for the development of *in vitro* lung tumor model using A549-MRC5 co-cultures. The PPMS had a robust and favorable architecture for cell attachment and proliferation to develop an *in vitro* lung tumor model. The co-cultures on PPMS showed greater chemoresistance to the tested anti-cancer drugs than co-cultures grown as a monolayer. These studies indicate the potential of the PPMS in the development of *in vitro* lung tumor models, which can be used for early, accurate screening of promising therapeutic agents, and for studying mechanisms of cancer progression. In the future, we intend to develop a more physiologically relevant multicellular model using human patient-derived lung cancer cells and also attempt to study the mechanism of chemoresistance mediated by the PPMS scaffold.

## Data Availability Statement

All datasets generated for this study are included in the article/Supplementary Material.

## Author Contributions

DD and DL contributed equally in conducting experiments and data analysis, writing, reviewing, and editing manuscript. RM helped in preparation of particles. AG helped in cell culture experiments. JM contributed by conceptualizing, mentoring, fund acquisition, and responsible for final editing of manuscript. All authors contributed to the article and approved the submitted version.

## Conflict of Interest

The authors declare that the research was conducted in the absence of any commercial or financial relationships that could be construed as a potential conflict of interest.

## Supplementary Material

File consists of EDS spectra of AMS, pore analysis, SEM images of A549 cells on PPMS and PLGAMS, cell seeding optimization experiment, Live dead of co-culture, anticancer screening for 24 h.
