# Supplementary material for: Porous Polymeric Microspheres With Controllable Pore Diameters for Tissue Engineered Lung Tumor Model Development
Source: Front Bioeng Biotechnol. 2020 Jul 10;8:799. doi: 10.3389/fbioe.2020.00799 (PMC7365955; doi:10.3389/fbioe.2020.00799)

Supporting information contains

Title	Page #
**Figure S1:** EDS spectra of AMS showing the presence of Ca^{2+} ion peak	**S2**
**Figure S2:** Pore analysis using Particle Analysis tool on ImageJ software	**S3**
**Figure S3:** SEM images of A549 cells on PPMS and PLGAMS, as well as morphologies of both particles following removal of cells by trypsinization	**S4**
**Figure S4:** Cell seeding optimization experiment based on the growth kinetics of A549 adenocarcinoma cells on PPMS (A) and PLGAMS (B)	**S5**
**Figure S5.** A549 and MRC-5 were seeded (1:1 ratio) at 25,000 cells/mg/well and live/dead assays were conducted at each time point before imaging under Nikon CLSM	**S6**
**Figure S6:** Comparative *in vitro* screening of anticancer drugs using WST-1 assays at 24h post-treatment	**S7**
**Figure S7.** Overlay of live/dead images of co-cultured monolayer MRC-5 and A549 (22,000 cells/well at 1:1 seeding ratio) after 6 hours of treatment	**S8**
**Figure S8.** Overlay live/dead images of cocultured MRC-5 and A549 on Col-PPMS (22,000 cells/mg/well at 1:1 seeding ratio) after 6 hours of treatment	**S9**
**Figure S9.** Overlay live/dead images of cocultured monolayer MRC-5 and A549 (22,000 cells/well at 1:1 seeding ratio) after 24 hours of treatment	**S10**
**Figure S10.** Overlay live/dead images of cocultured MRC-5 and A549 on Col-PPMS (22,000 cells/mg/well at 1:1 seeding ratio) after 24 hours of treatment	**S11**

Supplementary figures

Figure S1: EDS spectra of AMS showing the presence of Ca^{2+} ion peak

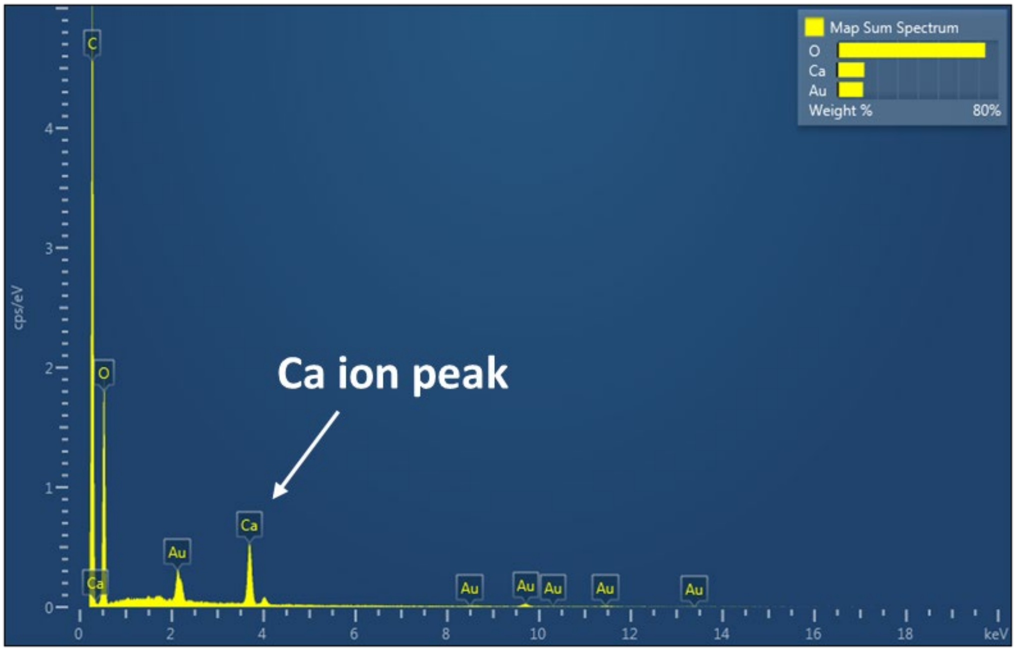

Figure S2: Pore analysis using Particle Analysis tool on ImageJ software. A) Masked picture after applying threshold. B) Pores detected with the analysis tool. Scale bar 100 µm

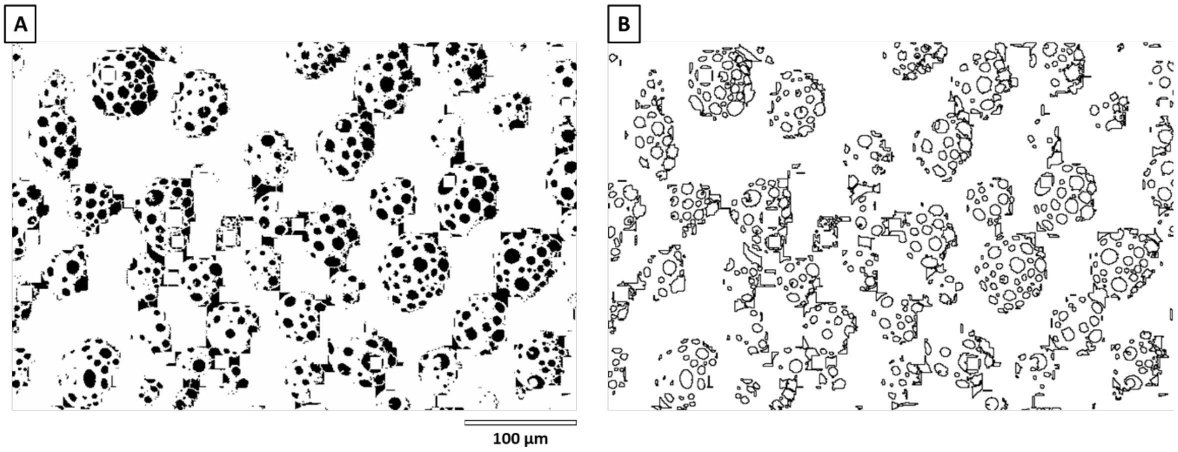

Figure S3: SEM images of A549 cells on PPMS and PLGAMS, as well as morphologies of both particles following removal of cells by trypsinization. Cell attachment doesn't seem to greatly influence degradation of the particles *in vitro*. PPMS shows clear pores until day 28 whereas PLGAMS shows shrunken morphology

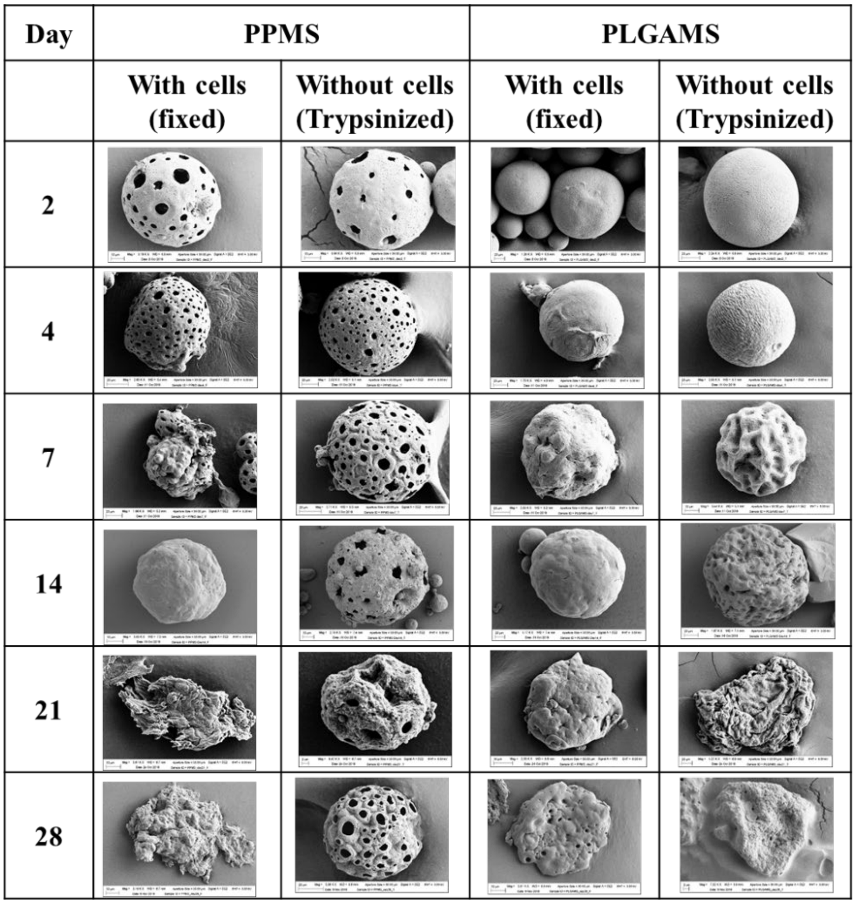

Figure S4: Cell seeding optimization experiment based on the growth kinetics of A549 adenocarcinoma cells on PPMS (A) and PLGAMS (B). Comparisons between both particles for each seeding density is given in Figure 4A-D. WST-1 assays were performed using standard protocol. Greater cell numbers were observed on PPMS. Seeding densities = 5×10^3, 10×10^3, ^{25} $\times 10^{3,}$ 50×10^3 cells/mg of microparticles/well. Data as mean ± SD, n = 3.

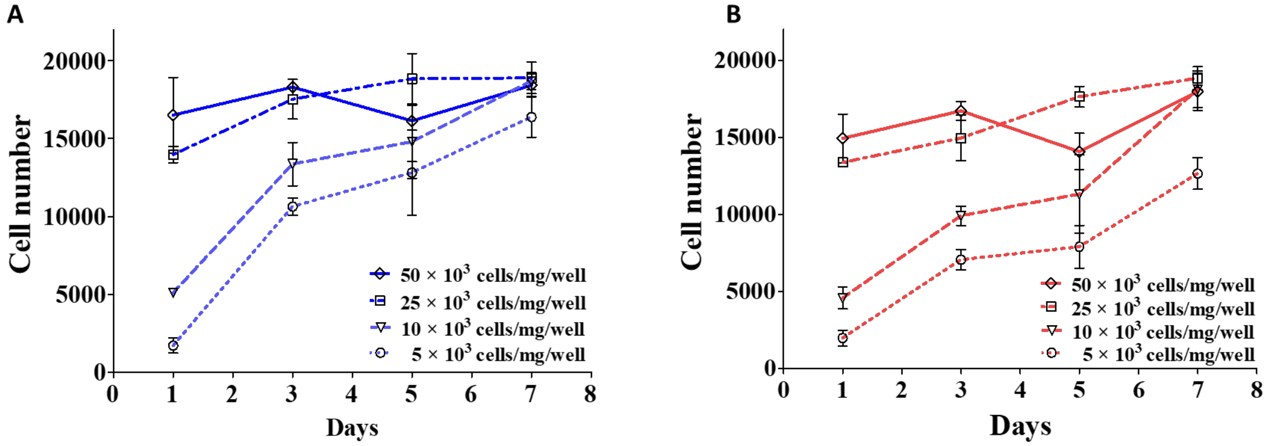

Figure S5. A549 and MRC-5 were seeded (1:1 ratio) at 25,000 cells/mg/well and live/dead assays were conducted at each time point before imaging under Nikon CLSM. Live cells were stained with Calcein AM and expressed green fluorescence while dead cells were stained with Ethidium homodimer and expressed red fluorescence. Pictures are overlay of red and green fluorescent images. Inset: phase contrast images of the same view. Scale bar 100 μm.

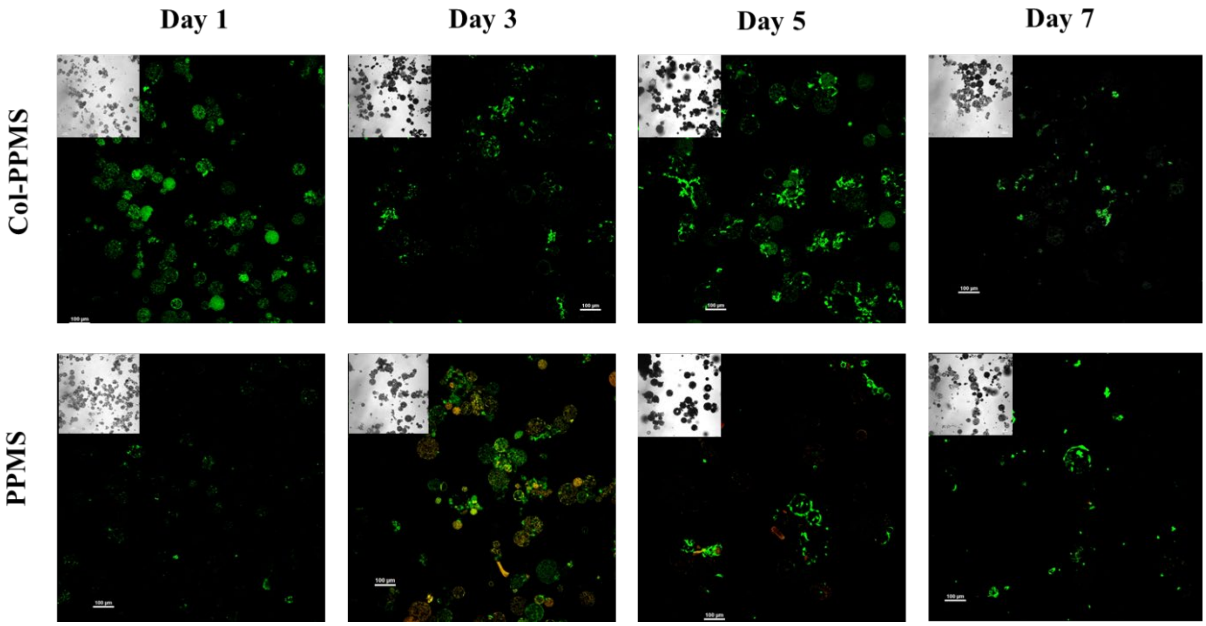

Figure S6: Comparative *in vitro* screening of anticancer drugs using WST-1 assays at 24h post-treatment. Percent cell viability was calculated as the ratio of absorbance (450 - 650nm) of sample with respect to the untreated control group. Application of student t-test showed a significant difference (*p<0.001, # p<0.01) in the responses of the monolayer co-culture and PPMS co-culture upon treatment with curcumin, cisplatin-etoposide combination, and cisplatin-gemcitabine combination.

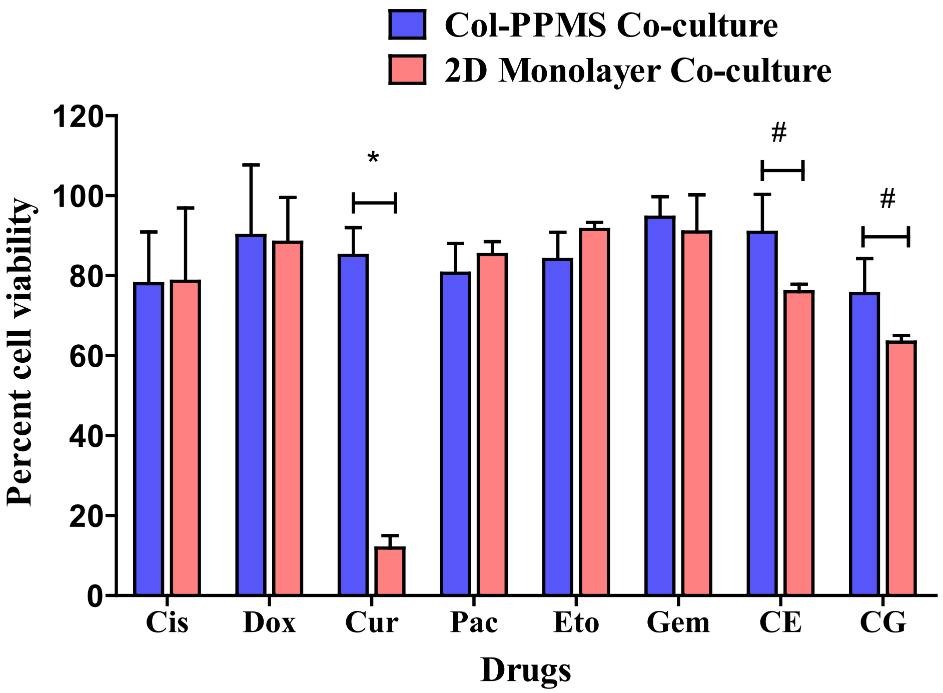

Figure S7. Overlay of live/dead images of co-cultured monolayer MRC-5 and A549 (22,000 cells/well at 1:1 seeding ratio) after 6 hours of treatment. Live cells were stained with Calcein AM and expressed green fluorescence while dead cells were stained with Ethidium homodimer and expressed red fluorescence. Images were captured on EVOS fluorescent microscope. Scale bar 400 μm

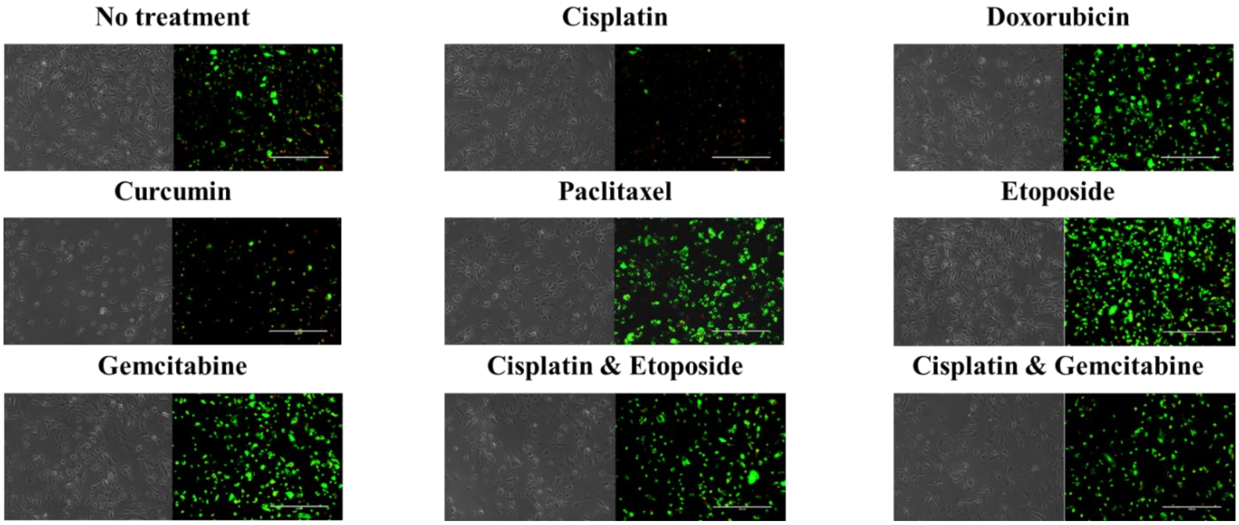

Figure S8. Overlay live/dead images of cocultured MRC-5 and A549 on Col-PPMS (22,000 cells/mg/well at 1:1 seeding ratio) after 6 hours of treatment. Live cells were stained with Calcein AM and expressed green fluorescence while dead cells were stained with Ethidium homodimer and expressed red fluorescence. Images were captured on EVOS fluorescent microscope. Scale bar 400 µm

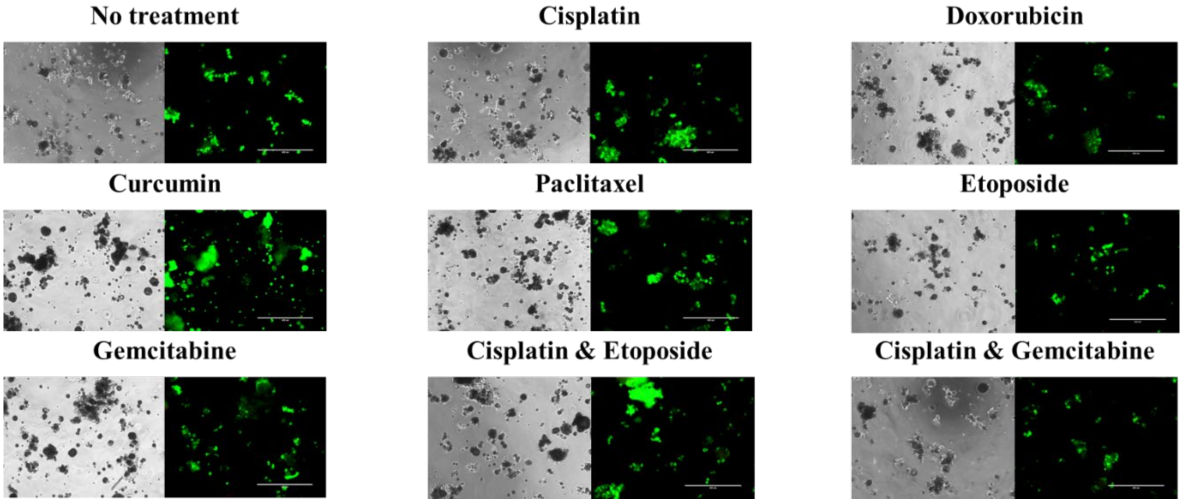

Figure S9. Overlay live/dead images of cocultured monolayer MRC-5 and A549 (22,000 cells/well at 1:1 seeding ratio) after 24 hours of treatment. Live cells were stained with Calcein AM and expressed green fluorescence while dead cells were stained with Ethidium homodimer and expressed red fluorescence. Images were captured on EVOS fluorescent microscope. Scale bar 400 μm

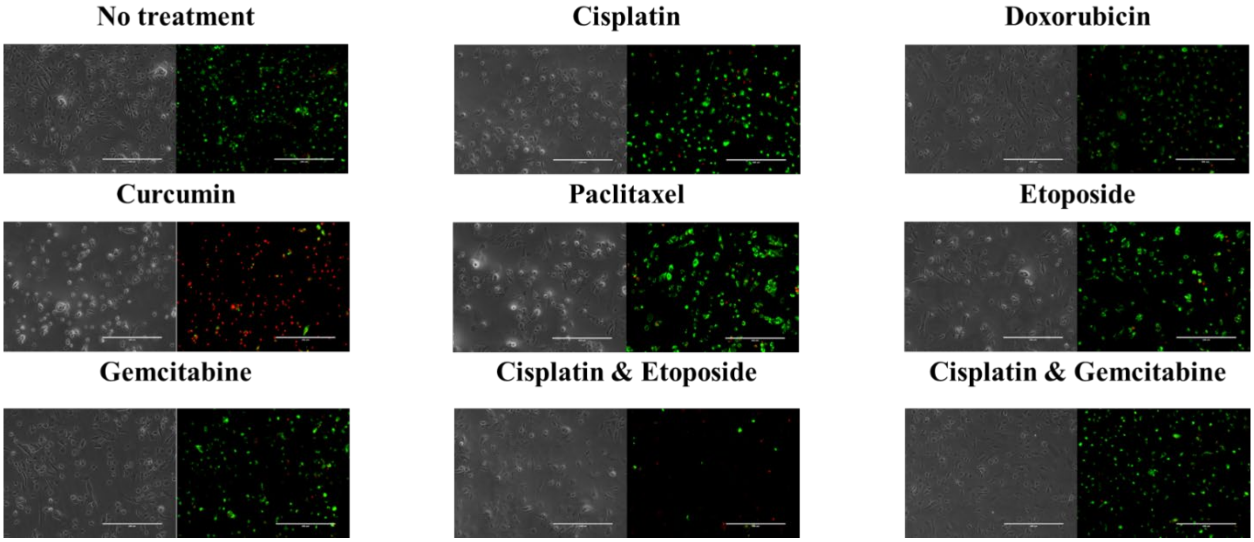

Figure S10. Overlay live/dead images of cocultured MRC-5 and A549 on Col-PPMS (22,000 cells/mg/well at 1:1 seeding ratio) after 24 hours of treatment. Live cells were stained with Calcein AM and expressed green fluorescence while dead cells were stained with Ethidium homodimer and expressed red fluorescence. Images were captured on EVOS fluorescent microscope. Scale bar 400 µm

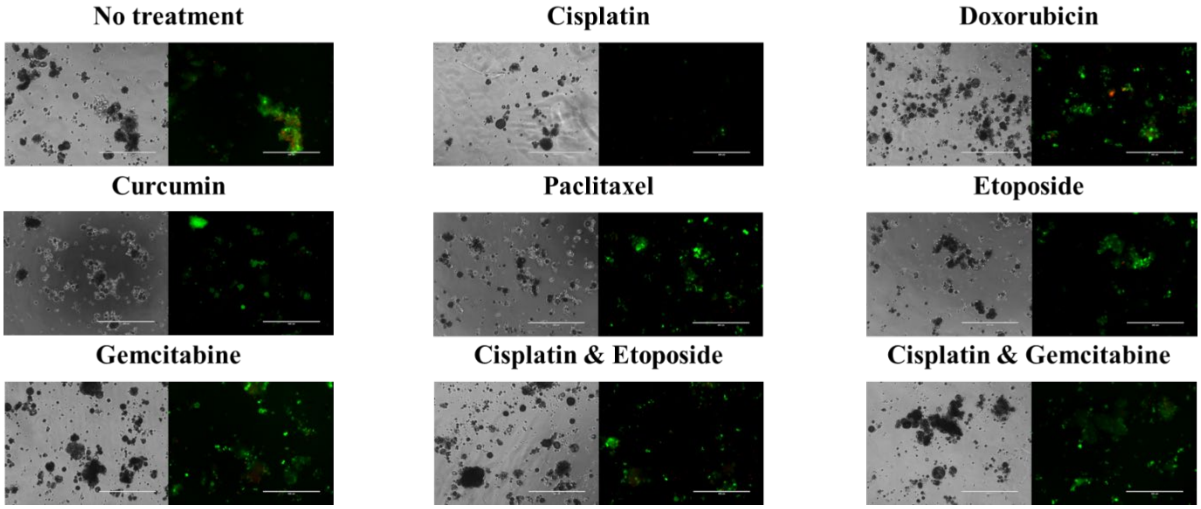

Supplement: Supplementary file 1 [file Image_1.pdf]
